# Old and new cluster designs in emergency field surveys: in search of a one-fits-all solution

**DOI:** 10.1186/1742-7622-5-7

**Published:** 2008-07-08

**Authors:** Oleg O Bilukha

**Affiliations:** 1 International Emergency and Refugee Health Branch, Division of Emergency and Environmental Health Services, National Center for Environmental Health, Centers for Disease Control and Prevention, 4770 Buford Hwy, Mailstop F-60, Atlanta, Georgia, 30341, USA

## Abstract

**Introduction:**

Cluster surveys are frequently used to measure key nutrition and health indicators in humanitarian emergencies. The survey design of 30 clusters of 7 children (30 × 7) was initially proposed by the World Health Organization for measuring vaccination coverage, and later a design of 30 clusters of 30 children (30 × 30) was introduced to measure acute malnutrition in emergency settings. Recently, designs of 33 clusters of 6 children (33 × 6) and 67 clusters of 3 children (67 × 3) have been proposed as alternatives that enable measurement of several key indicators with sufficient precision, while offering substantial savings in time. This paper explores expected effects of using 67 × 3, 33 × 6, or 30 × 7 designs instead of a "standard" 30 × 30 design on precision and accuracy of estimates, and on time required to complete the survey.

**Analysis:**

The 67 × 3, 33 × 6, and 30 × 7 designs are expected to be more statistically efficient for measuring outcomes having high design effects (e.g., vaccination coverage, vitamin A distribution coverage, or access to safe water sources), and less efficient for measuring outcomes with more within-cluster variability, such as global acute malnutrition or anemia. Because of small sample sizes, these designs may not provide sufficient levels of precision to measure crude mortality rates. Given the small number (3 to 7) of survey subjects per cluster, it may be hard to select representative samples of subjects within clusters.

The smaller sample size in these designs will likely result in substantial time savings. The magnitude of the savings will depend on several factors, including the average travel time between clusters. The 67 × 3 design will provide the least time savings. The 33 × 6 and 30 × 7 designs perform similarly to each other, both in terms of statistical efficiency and in terms of time required to complete the survey.

**Conclusion:**

Cluster designs discussed in this paper may offer substantial time and cost savings compared to the traditional 30 × 30 design, and may provide acceptable levels of precision when measuring outcomes that have high intracluster homogeneity. Further investigation is required to determine whether these designs can consistently provide accurate point estimates for key outcomes of interest. Organizations conducting cluster surveys in emergency settings need to build their technical capacity in survey design to be able to calculate context-specific sample sizes individually for each planned survey.

## Introduction

Over the past three decades, field surveys using a cluster sample design have become a standard, popular, and widely used method of measuring key nutrition and health indicators in humanitarian emergencies [1-4]. This method was first widely popularized by the Expanded Program on Immunization (EPI) of the World Health Organization, which since 1978 has promoted using a 30 cluster design, with 7 children per cluster (popularly known as "30 × 7") for measuring immunization coverage [5,6]. The field applications of this method expanded rapidly to include measuring such key emergency indicators as acute malnutrition, crude and under-5 mortality, cumulative incidence of diarrhea and respiratory disease, access to safe water, prevalence of physical signs of micronutrient deficiencies, and several others [7-10]. The challenges of measuring these indicators with sufficient precision triggered modifications to the initially proposed standard 30 × 7 design and led to the introduction of a recommendation for use of a 30 clusters of 30 children (30 × 30) design for measuring acute malnutrition in cluster surveys [11,12]. Recently published field survey manuals [13,14] advocate calculating sample sizes and determining the number of clusters individually for each planned survey. This methodology would allow for achieving the required level of precision while keeping the sample size and the number of clusters to a necessary minimum, thus providing for rational and efficient use of scarce time and resources in emergency settings. Such calculations take into account not only statistical variables, such as the expected prevalence of the indicators being measured and the expected level of clustering of these indicators, but also logistics and the time required to survey households or individuals and to move from cluster to cluster. These calculations require substantial specialized expertise, which often is not available to organizations conducting surveys in emergencies [15-18]. The search, therefore, continues for the "standard" designs that would enable measurement of most of the key emergency indicators with acceptable precision while requiring less time and fewer resources than a 30 × 30 design. Most recently, 33 × 6 and 67 × 3 cluster survey designs have been proposed as alternatives that allow for measuring several indicators with sufficient precision, while offering substantial savings in cost, time, and manpower [19,20].

In this paper, we will explore the expected statistical effects on precision of using the newly proposed 33 × 6 and 67 × 3 designs as well as the "old" 30 × 7 EPI design, as compared with the conventional 30 × 30 design. We will then discuss the situations and outcomes for which these designs may be most statistically efficient (i.e., may result in the least loss of precision compared to a 30 × 30 design); offer some thoughts on the potential of these designs to introduce bias into the prevalence estimates; and explore situations where these designs can offer the most substantial savings of time and resources.

## Analysis

### Achieving acceptable precision

A simplified formula used for calculating the width of the one side of the two-sided 95% confidence interval in cluster surveys is [14]:

d = Z*sqrt(p(1-p)*DEFF/n)

where d = the width of one side of the two-sided 95% confidence interval

n = sample size

p = the prevalence of the outcome being measured

DEFF = design effect

Z = z value (generally 1.96)

The design effect is the ratio of the variance of the estimate under the actual (for example, cluster) design to the variance of the estimate assuming that the same data have been collected by simple random sampling [3,10]. By changing the cluster design, we can modify both the design effect (DEFF) and the sample size (n), while Z and p will not be affected. The design effect can be presented as a function of the average cluster size (m) and the intracluster correlation coefficient (Rho), which describes the relatedness of cluster data by comparing the variance within clusters with the variance between clusters [21,22]:

DEFF = 1 + Rho(m-1)

Therefore, the smaller the cluster size, the smaller the design effect. Decreasing the design effect through decreasing the cluster size may, to a certain degree, offset the loss of precision resulting from decreasing the overall sample size [3]. For example, if we observed a design effect of 2 for a certain indicator in a 30 × 30 survey, then Rho for this indicator in the surveyed population can be calculated as:

Rho = (DEFF - 1)/(m-1) = (2-1)/(30-1) = 0.0345

Using this value of Rho, we can then calculate an expected DEFF for 33 × 6, 67 × 3, and 30 × 7 designs:

DEFF_33 × 6 _= 1 + 0.0345*(6-1) = 1.1725

DEFF_67 × 3 _= 1 + 0.0345*(3-1) = 1.0690

DEFF_30 × 7 _= 1 + 0.0345*(7-1) = 1.2070

As can be seen, expected design effects for these three designs are substantially lower than the design effect of 2 observed in a 30 × 30 survey.

We can now calculate the expected relative change in the width of the confidence interval of the 33 × 6 design compared to the 30 × 30 design as a ratio of sqrt(DEFF_33 × 6_/n_33 × 6_) to sqrt(DEFF_30 × 30_/n_30 × 30_), since p and Z do not change:

sqrt(1.1725/198)/sqrt(2/900) = 0.07695/0.04714 = 1.63

Therefore, the 95% confidence interval in the 33 × 6 design is expected to be 63% wider than the 95% confidence interval in a 30 × 30 design.

Table 1 presents similar calculations showing changes in precision for the three designs (67 × 3, 33 × 6, and 30 × 7) compared to a 30 × 30 design for design effects of different magnitude. As can be seen, all three designs resulted in much lower design effects than a 30 × 30 design. Nevertheless, at lower design effects (1.25–2.0 in 30 × 30 design), the expected loss in precision (or increase in the width of the confidence interval) was substantial: the width of the confidence intervals increased by 55%–95% compared with the confidence interval of a 30 × 30 design. This loss of precision decreased with increasing design effect. Of the three designs, the 67 × 3 design consistently resulted in the lowest loss of precision and actually out-performed the 30 × 30 design at higher design effects (7 and above). The 30 × 7 design performed similarly to the 33 × 6 design.

**Table 1:** Expected change in precision for 67 × 3, 33 × 6 and 30 × 7 designs compared with 30 × 30 design

30 × 30 design Observed DEFF	Rho	67 × 3 design		33 × 6 design		30 × 7 design	
		
		Expected DEFF	Change in the width of the CI,^1 ^ %	Expected DEFF	Change in the width of the CI,^1 ^ %	Expected DEFF	Change in the width of the CI,^1 ^ %
1	0	1	+111.6	1	+113.2	1	+107.0
1.25	0.0086	1.0172	+90.9	1.0431	+94.7	1.0517	+89.9
1.5	0.01724	1.0345	+75.7	1.0862	+81.4	1.1034	+77.6
2	0.03448	1.0690	+54.7	1.1724	+63.2	1.2069	+60.8
2.5	0.05172	1.1034	+40.6	1.2586	+51.3	1.3103	+49.9
3	0.06897	1.1379	+30.3	1.3448	+42.7	1.4138	+42.1
4	0.10345	1.2069	+16.2	1.5172	+31.3	1.6207	+31.8
5	0.13793	1.2759	+6.9	1.6897	+23.9	1.8276	+25.2
7	0.20690	1.4138	-4.9	2.0345	+14.9	2.2414	+17.1
10	0.31034	1.6207	-14.8	2.5517	+7.7	2.8621	+10.8

^1 ^Relative expected change in the width of the 95% confidence interval compared to the 30 × 30 design

**Figure 1:**
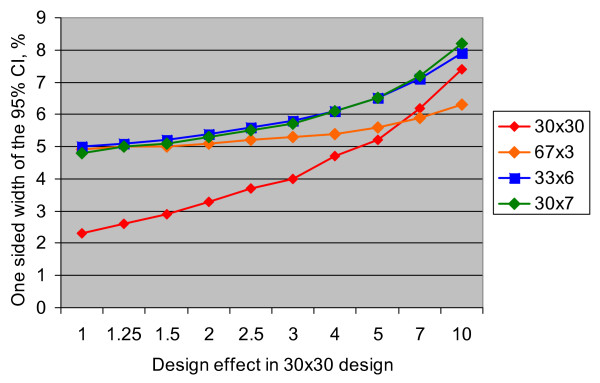
The width of the 95% confidence interval for different cluster designs given 15% prevalence of the measured outcome.

Figure 1 presents the half-width of the 95% confidence interval at different design effect levels in scenarios where the prevalence of the measured outcome is 15%. Again, it can be seen that the losses of precision for all three designs, compared to the 30 × 30 design, are the largest at the lowest design effect levels, and they decrease with increasing design effect. Performance of the 30 × 7 design is similar to that of the 33 × 6, and the 67 × 3 design is clearly superior at the highest design effect levels. Although for other prevalence levels of the measured outcome the absolute width of the confidence intervals will differ from those shown in Figure 1, the relative expected differences in precision among different designs will remain the same, as shown previously in Table 1.

In practical terms, these results mean that 67 × 3, 33 × 6, and 30 × 7 designs are most statistically efficient for measuring outcomes or indicators that have high intracluster correlation, and that they are not as statistically efficient (resulting in substantial loss of precision compared to that of the 30 × 30 design) for outcomes with a low degree of clustering. Unfortunately, the body of literature that provides guidance on design effects for different outcomes measured in cluster surveys in emergencies is very limited, and design effects are not routinely included in survey reports. Two recent papers [3,20] provide, however, some insight into commonly seen magnitudes of design effects for indicators frequently measured in emergencies. From these papers, it seems that the prevalence of global acute malnutrition and anemia tend to have the lowest design effects (on the magnitude of 1.2–2.0). Somewhat higher design effects (around 2.5–4.0) are observed for a cumulative 2-week incidence of diarrhea and acute respiratory infection (ARI), and yet higher design effects (5.0 and higher) are observed for vitamin A distribution and vaccination coverage. The highest design effects (10 and higher) are likely to be observed for some household-level indicators, such as access to potable water or to latrines [20]. Therefore, 67 × 3, 33 × 6, and 30 × 7 designs would be most efficient for measuring the most clustered outcomes, such as access to potable water and latrines, vaccination and vitamin A distribution coverage; moderately efficient for measuring cumulative 2 week incidence of ARI or diarrhea; and least efficient for measuring outcomes with the most within-cluster variability, such as global acute malnutrition and anemia.

As a result of small overall sample sizes (around 200 households), 67 × 3, 33 × 6, and 30 × 7 designs are not likely to provide sufficient levels of precision to measure crude or under-5 mortality rates. For example, assuming that the true under-5 mortality rate in the population is 1.0 per 10,000 per day, a recall period of 3 months, and on average 1 child under 5 years of age per household, the expected number of under-5 deaths detected in the 33 × 6, 30 × 7, or 67 × 3 surveys is only about 1.8. This will result not only in a wide confidence interval, even if DEFF for mortality is low, but also in unstable point estimates.

It is also important to consider the minimum desired level of precision in each concrete situation. For example, if in the scenario presented in Figure 1 the investigators are measuring an outcome with an expected design effect of 2 in a 30 × 30 design, and consider that achieving the precision of ± 6% is sufficient, they could probably use one of the three designs (67 × 3, 33 × 6, and 30 × 7). If, however, a higher level of precision is required, then the cluster design with the larger overall sample size would need to be considered. In addition, these designs may not provide sufficient precision for the subgroup estimates within the survey sample – for example, when separate estimates are desired for males and females or for different age subgroups within the sample of children under 5 years of age.

### Avoiding threats to validity

One important question is whether designs in which the number of survey subjects per cluster is low (3 in 67 × 3 to 7 in 30 × 7) can reliably ensure that the subjects sampled in each cluster are representative of that cluster. With only 3 survey subjects per cluster, such representativeness may be hard to achieve, especially if the EPI method, rather than random sampling, is used for selecting households or subjects within a cluster [14]. It is conceivable that teams that either are pressed for time (if they are expected to complete several clusters in one day), poorly trained, or poorly motivated would be tempted to select 3 or 6 most conveniently or centrally-located houses to achieve the sample size for a given cluster. For some indicators, such "central" bias in sampling may result in biased prevalence estimates. With the 30 × 30 design, the teams would often have the whole day to complete one cluster and may be inclined to better adhere to selection procedures prescribed by the EPI method, thus achieving better representativeness. These problems could be overcome to a certain degree if random selection procedures, rather than the EPI method, are used for subject selection. These procedures, however, often require substantial additional time and effort to map or enumerate households within a cluster before random selection can be carried out.

One way to assess the seriousness of this problem is to compare the prevalence estimates obtained from a 67 × 3 or a 33 × 6 survey to those obtained by using a 30 × 30 design, given that the same population is sampled. One recent paper [19] presents such an opportunity. In that paper, 67 × 3, 33 × 6, and 30 × 30 surveys, all measuring the same outcomes, were conducted in the same population. Prevalence estimates for global acute malnutrition, stunting, underweight, and vaccination coverage were roughly comparable across the three designs. The prevalence of severe acute malnutrition obtained in the 67 × 3 survey (5.7%) was, however, more that 2.5 times higher than that obtained in 33 × 6 and 30 × 30 surveys (2.1% and 2.3%, respectively). More interestingly, the 2-week cumulative incidence of diarrhea and fever measured by 33 × 6 (31.2% and 30.7%, respectively) and 67 × 3 (32.5% and 37.5%, respectively) surveys were substantially higher than estimates for diarrhea and fever obtained from a 30 × 30 survey (24.4% and 24.8%, respectively). One possible (albeit admittedly speculative) explanation for these differences may be that subjects in 33 × 6 and 67 × 3 surveys were more likely to be sampled in central locations of the clusters, where higher population density may result in higher incidence of common infections in children than in peripheral locations. Therefore, it would be important to conduct additional, similar studies to assess whether the point estimates for some commonly measured indicators in 33 × 6 or 67 × 3 surveys consistently and substantially deviate from the estimates obtained in 30 × 30 surveys and to explore potential reasons if such differences are found. Careful training and supervision of teams conducting these surveys, and use of random rather than EPI procedures for subject or household selection within clusters, will need to be emphasized.

### Saving time and resources

One attractive feature of the survey designs discussed in this paper is the dramatically reduced sample size (from 900 in a 30 × 30 survey to around 200). Time and resource savings resulting from this more than four-fold decrease in sample size will likely be one of the main arguments for wider field application of these designs.

In a simplified form, the time needed for a team to complete a survey in the field (assuming that teams do not need to return to their base at the end of each day) can be expressed as:

T = k*t_1 _+ (k+1)*t_2 _+ n*t_3_

where:

T – total time to complete a survey for one team

t_1 _– average time needed for initial introduction of team to the community in each cluster and initial selection of households within cluster

t_2 _– average time of travel between clusters or between cluster and base

t_3 _– average time to complete survey for one survey subject/household, including travel between houses

k – number of clusters

n – total sample size

**Figure 2:**
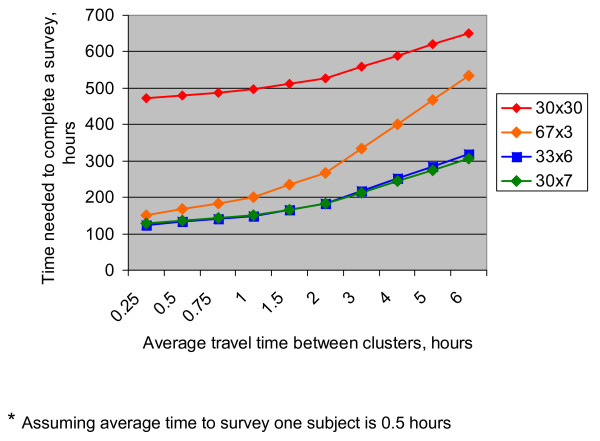
Time needed to complete a survey for different cluster designs depending on average travel time between clusters.

Mathematically, it can be shown that relative time savings in 67 × 3, 33 × 6, and 30 × 7 designs, compared to 30 × 30 design, increase with increasing t_3 _and decrease with increasing t_1 _and t_2_. Since the travel time is likely to vary the most from survey to survey, it would be interesting to explore how the time savings change, depending on travel time. Figure 2 presents total time to complete the surveys of different designs for different average travel times (t_2_), varying from 15 minutes to 6 hours (assuming both t_1 _and t_3 _to be 30 minutes). As can be seen, the biggest time savings (3 to 4 times less than for the 30 × 30 design) are expected when the average travel times between clusters are small (15 minutes to 1 hour). With increasing average travel time between clusters, relative time savings decrease, especially for the 67 × 3 design, which at longer travel times (5–6 hours) offers little time advantage over the 30 × 30 design. For longer travel times, the 67 × 3 cluster design may present an additional disadvantage of higher fuel costs, since the teams need to travel long distances to 66 instead of 30 different locations. The 33 × 6 and 30 × 7 designs produce very similar time savings, and at longer travel times (4–6 hours) they would still require less than half the time needed to complete a 30 × 30 survey. Therefore, in situations where travel times between clusters are long, logistically 33 × 6 and 30 × 7 designs would be preferable to the 67 × 3 design.

It should be emphasized, however, that the scenario presented above is fairly simple in that it assumes that the team does not need to return to the base at the end of each day and can spend nights in the field, either in or between the clusters it is surveying. Also, it assumes that introduction of the team to the community and initial household selection within a cluster are not very time-consuming. In cases when mapping or enumeration of the households within each cluster needs to be carried out as part of random selection procedures, this initial phase will likely take much longer than 30 minutes. The quality of roads in the survey area and vehicles available to survey teams may have a substantial impact on travel time. Therefore, a time estimation exercise that takes into account specific circumstances of each planned survey needs to be carried out in the planning phase so as to evaluate expected time requirements associated with each of the alternative designs.

## Conclusion

Overall, cluster designs with substantially decreased cluster size and overall sample size, such as the 67 × 3, 33 × 6, and 30 × 7 designs discussed in this paper, may offer substantial time and cost savings compared to the traditional 30 × 30 design, and they may provide levels of precision that are acceptable (or at least comparable to the 30 × 30 design) for measuring some common outcomes of interest in humanitarian emergencies. This is particularly the case for outcomes that tend to have high intracluster homogeneity, such as vaccination coverage, vitamin A supplementation coverage, or access to safe water. Using these designs to measure outcomes that are normally less clustered (e.g, global acute malnutrition) may result in a sizeable loss in precision (resulting in confidence intervals 1.5 to 2 times wider than those obtained with the 30 × 30 design), and may therefore need to be carefully considered vis-à-vis context-specific minimum requirements for precision. Because of the small overall sample size, these designs are not likely to provide sufficient precision to measure crude or under-5 mortality rates. The question of the ability of these designs to consistently provide accurate (or unbiased) point estimates for key indicators routinely measured in humanitarian emergencies remains open, and it is subject to further investigation. Good training and supervision of the teams implementing these designs in the field and use of random rather than EPI methods for household selection will be important for ensuring selection of representative samples within clusters.

Because of substantially reduced overall sample size, these designs will likely result in substantial (2–4 times compared to 30 × 30 design) time savings. The magnitude of these time savings will depend on several factors, including the average time needed to complete a survey for one survey subject or household, the average time to select households within a cluster, and the average time needed to travel between clusters. The 67 × 3 design will provide the least time savings relative to the 30 × 30 design, and it may be logistically difficult to implement if travel distances between clusters are large. The 33 × 6 and 30 × 7 designs are expected to perform similarly to each other, both in terms of statistical efficiency and in terms of time required to complete the survey.

Finally, despite several proposed "standard" designs that can provide the minimum required level of accuracy and precision, some of which were discussed in this paper, the best solution is to calculate sample sizes individually for each planned survey, taking into account the specific indicators being measured, context-specific levels of precision required, logistic and security concerns, availability of suitable personnel, and other factors. Many surveys in emergencies strive to measure multiple outcomes, including nutritional status, mortality, water and sanitation indicators, access to feeding programs and food aid, among others. In these situations, sample sizes to achieve acceptable levels of precision need to be calculated for each of the key indicators [14], and the best solution in terms of sampling design and sample size needs to be determined based on this information. It is important to continue building technical capacity in cluster survey design within organizations conducting cluster surveys in humanitarian emergencies [15-17]. Several important initiatives (Standardized Monitoring and Assessment of Relief and Transitions (SMART), Health and Nutrition Tracking Service) and recently published survey manuals [13,14] should prove instrumental in achieving this goal.

## Competing interests

The author declares that he has no competing interests.

## Authors' contributions

OOB designed the study, performed analysis, wrote and revised the manuscript.
